# Effect of Phase Heterogeneity on the Properties of Poly(vinyl alcohol)-Based Composite Pervaporation Membranes

**DOI:** 10.3390/membranes12121185

**Published:** 2022-11-24

**Authors:** Svetlana V. Kononova, Roman V. Kremnev, Galina N. Gubanova, Elena N. Vlasova, Elena N. Popova, Milana E. Vylegzhanina, Anatoly Ya. Volkov

**Affiliations:** Institute of Macromolecular Compounds, Russian Academy of Science, Bolshoy pr. 31, 199004 Saint-Petersburg, Russia

**Keywords:** microphase separation, pervaporation, carbon nanotubes, poly(vinyl alcohol), poly(acrylic acid), poly(N,N-dimethylaminoethyl methacrylate)

## Abstract

The structure, thermophysical characteristics, and pervaporation properties of composite membranes based on poly(vinyl alcohol) (PVA) are studied in dependence of the film preparation conditions. It is shown that the nature of the supramolecular organization of the composite polymer film determines which of the components of the separated mixtures of toluene and heptane predominantly penetrate through the corresponding pervaporation membrane. The observed structural effects can become more pronounced if the second component of a polymer mixture is purposefully selected (in this case, poly(N,N-dimethylaminoethyl methacrylate) instead of poly(acrylic acid)) or a nano-sized filler that can be well dispersed in the polymer matrix is introduced. Multi-wall carbon nanotubes are introduced into binary PVA-containing polymer blends. The influence of these fillers on the structure and transport properties of the obtained membranes is studied.

## 1. Introduction

Poly(vinyl alcohol) (PVA) is a synthetic water-soluble and semi-crystalline polymer produced industrially worldwide [1]. This polymer has unique characteristics, including good processability, hydrophilicity, biocompatibility, film-forming ability, non-toxicity, biodegradability, and selective transport properties. Owing to these favorable features, PVA finds applications in various areas of science and technology; it is used in the development of drug delivery systems and biocompatible glucose sensors [2], in water purification [3], and in the preparation of antibacterial active substances [4,5,6,7,8] and membranes. Poly(vinyl alcohol) is used in various forms (powders, fibers, films, etc.).

**Figure 1:**
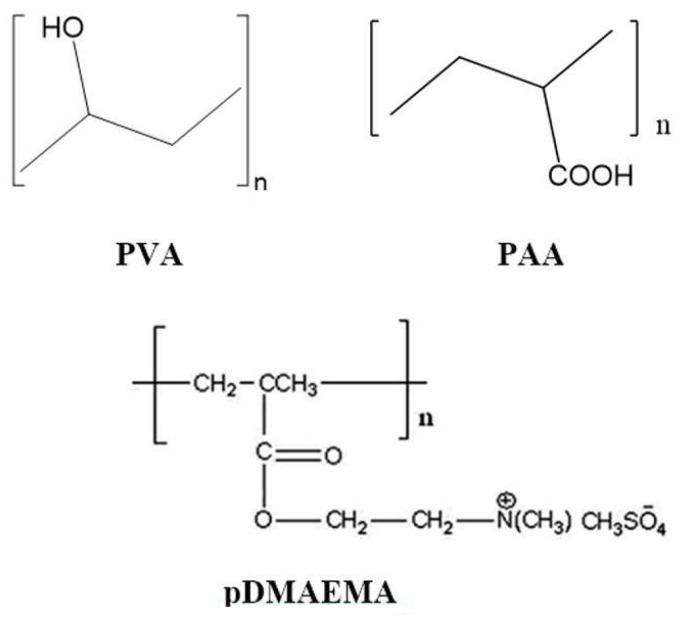
Chemical structures of the studied polymers.

PVA is a polymer that is synthesized by the alkaline/acid hydrolysis of polyvinyl esters, primarily poly(vinyl acetate). The application areas of this polymer are determined by its hydrophilicity, as well as by the presence of acetate groups that have not entered into the hydrolysis reaction. The availability of hydroxyl groups in the macromolecule (as shown in Figure 1 ensures the solubility of PVA in water and its use in polar solvents on the one hand, and the possibility of its functionalization on the other. In particular, it is possible to create intra- and intermolecular bonds and to obtain PVA-containing intermolecular complexes.

The crystallinity of PVA is also related to the existence of a large number of hydroxyl groups in the polymer. Additionally, the crystallinity of the polymer is influenced by the prehistory of a sample, the presence of branching, the degree of hydrolysis, and the distribution of the residual acetate groups along the chain. The higher the degree of hydrolysis, the higher the crystallinity of a PVA sample.

To disrupt the intermolecular and intramolecular interactions existing between the hydroxyl groups in PVA chains, the polymer is subjected to modification. On the other hand, the products of PVA crosslinking via hydroxyl groups find numerous applications in scientific and industrial practice [9,10]. Crosslinking with dialdehydes or other substances [11,12] completely changes the physical and chemical properties of the polymer [13]. Many researchers consider it necessary to expand the application potential of these PVA derivatives [14,15]. PVA is modified by introducing reactive functional groups, such as carboxyl, sulfonate, and amino groups, or by producing interpenetrating networks (IPNs), which impart the properties of a second polymer component to the PVA-based material [16]. All of these methods make it possible to obtain structurally complex composite materials on the basis of poly(vinyl alcohol). 

The ability of PVA to undergo chemical and physical transformations is one of the reasons for its widespread use as a membrane-forming polymer. Since PVA is able to react with other polymers at the expense of hydroxyl groups present in its structure, various polymer–polymer PVA-containing composite membranes have been developed [17]. If the second polymer contains functional fragments capable of interacting with hydroxyl groups, binary PVA-based composites are conventionally considered as crosslinked systems. Although these types of composite materials are described in detail in the scientific literature, their behavior is not well understood; they may exhibit properties that cannot be predicted based on the preliminary information about the individual components. 

This effect can be explained by the multifactor nature of the systems under discussion. The interaction with another polymer resulting in the formation of an interpolymer complex can lead to a significant change in the properties of PVA. For example, this process can affect not only the hydrophilicity of the polymer, but also its intermolecular organization (including crystallinity). Among the most interesting manifestations of this effect are the pervaporation properties of the composite membranes. Bearing in mind the high hydrophilicity of PVA, many experts have logically developed PVA-based pervaporation membranes for the extraction of water from water–organic mixtures [13,18,19,20]. The selective transport properties of the PVA-containing composite membranes are targeted and changed by shifting the hydrophilic–lipophilic balance in these systems. 

At the same time, there are studies that describe composite PVA-containing membranes that are effective in the processes of organophilic pervaporation. Predictable examples of the separation of alcohol-containing mixtures of liquids due to the formation of short-lived interactions between the hydroxyl groups of PVA and the hydroxyl groups of transported alcohols are known. This leads to the preferential transport of polar liquids capable of forming hydrogen bonds through PVA-containing hydrophilic membranes. However, as is known, an increase in separation selectivity is achieved in such systems by limiting the swelling of the membrane in the predominantly transported component. Thus, selective transmembrane transport by a facilitated mechanism is achieved by forming composites containing structurally organized regions that limit the swelling of the membrane material in the target transported substances. 

Even the most complex pervaporation separations can be carried out in this way, such as the selective transport of components of mixtures of aromatic and aliphatic hydrocarbons [21,22,23,24,25,26]. 

The separation of mixtures of aromatic and aliphatic hydrocarbons, as well as their isomers, such as benzene and cyclohexane, toluene and n-heptane, or toluene and iso-octane, has been intensively studied in recent years [27,28], but some issues remain unresolved. These problems can be solved using highly energy-intensive processes, such as rectification, azeotropic distillation, extractive distillation, and solvent extraction. In this case, the distillation method is not applicable for separating mixtures of liquids with close boiling points (for example, the boiling point of toluene is 111 °C, while the boiling point of n-heptane is 98 °C), meaning pervaporation is then a good alternative. 

As is known, the separation efficiency of a material in pervaporation processes is characterized by the separation factor, which is determined by the diffusion and sorption components. The sorption component of separation in the absence of specific interactions between a penetrant and a polymer (for example, in the case of poly(vinyl alcohol) and mixtures of toluene and heptane) is determined by the difference between the boiling points of the separated components. The diffusion component of the separation factor is controlled by the ratio between the sizes of the molecules of the separated penetrants and by the “rigidity of the ensemble of macromolecules”. The greater the difference between the sizes of penetrant molecules and the more rigid the ensemble of macromolecules, the higher the diffusion component of the separation factor [29]. Thus, the development of PVA-based selective membranes involves methods that allow one to regulate the packing of its macromolecules.

By controlling the amount of acetate groups, the authors [30] changed the intermolecular packing of the PVA (which, in fact, was a copolymer of PVA with poly(vinyl acetate)) and managed to increase the benzene permeability of the prepared membrane. The physical modification of the poly(vinyl alcohol) included the use of various fillers, e.g., calixarenes and cyclodextrins; this modification method led to the formation of two-phase systems [21,26,30]. In this way, it was possible to achieve the predominant transport of benzene; however, the permeability decreased by more than an order of magnitude in comparison with that of a pure poly(vinyl alcohol) membrane. An attempt was made to modify poly(vinyl alcohol) by mixing it with chitosan and filling the binary polymer matrix with graphite; the goal was to improve the pervaporation properties of the hybrid membrane during the separation of a benzene–cyclohexane mixture [31,32,33]. The membrane was investigated using scanning electron microscopy, Fourier IR spectroscopy, X-ray diffractometry, and dynamic mechanical analysis. The studies revealed a uniform distribution of graphite particles within the polymer matrix, an increase in the number of hydrogen bonds, a significant decrease in the degree of polymer crystallinity, an improvement of the mechanical properties of the membrane, and a significant increase in its free volume. Pervaporation studies involve varying the graphite content and the ratio between the poly(vinyl alcohol) and chitosan in the film. Thus, the modification of PVA by mixing it with another polymer or using a carbon filler results in a significant increase in the separation factor value. The use of carbon molecular sieves (CMS) makes it possible to reduce the number of intermolecular hydrogen bonds between poly(vinyl alcohol) chains, which leads to a decrease in the degree of crystallinity of the PVA membrane and the formation of an additional free volume [34]. Because the packing of the polymer becomes less dense, the permeability of the CMS-modified membranes increases; meanwhile, the effect of the swelling becomes more pronounced. Therefore, as the benzene content in the initial mixture and the separation temperature increase, the permeability of the modified membrane rises and the separation factor declines. 

Considering the above effects, the most interesting materials are composites of poly(vinyl alcohol) with poly(acrylic acid). Here, both polymeric components contain functional groups capable of forming hydrogen bonds. Research on PVA-PAA composites began many years ago, when interest was first aroused in the development of pervaporation membranes for various purposes [35,36,37]. These studies have been continued to the present day [38,39]. There are several significant reasons for the ongoing interest in this polymer pair. First, PVA is able to react with PAA (which contains carboxyl groups) due to the presence of hydroxyl groups in its structure [17]. Depending on the preparation conditions, it is possible to create a network of hydrogen bonds or to form ester bonds via esterification reactions [17]. By varying the quantitative ratio of the functional groups of the reacting polymers, one can produce composites with a set of new structural characteristics, and as a consequence new mechanical, thermophysical, and membrane properties.

It should be noted that (meth)acrylic polymers are also widely applied in the development of pervaporation membranes. Since esters of acrylic and methacrylic acids swell strongly in aromatic solvents, copolymers of esters with acids are used to separate aromatic and aliphatic hydrocarbons. Acrylic acid was chosen as a co-monomer to alkyl acrylates, because carboxyl groups of the copolymer can react with polyfunctional molecules to form crosslinked structures. As a result, the copolymers have flexible segments (acrylic groups), while crosslinking causes the appearance of a rigid phase [40,41,42]. In fact, the formation of intermolecular ester bonds upon the interaction between PVA and PAA should lead on the one hand to the formation of an “ester phase” that swells in aromatic solvents, and on the other hand to stiffening of the material due to crosslinking. Thus, the result is determined by a combination of two opposite factors. 

An analysis of the literature and the results of our preliminary studies showed that the discussed multifactorial systems are extremely difficult to predict. Studying them is like researching a “black box”. To obtain reliable basic information, it is necessary to study “simplified” composites first. In this regard, the present work was devoted to investigations of composite non-crosslinked PVA-based films and their behavior as pervaporation membranes. The film preparation conditions precluded the possibility of chemical reactions with the formation of ester groups.

## 2. Experimental

### 2.1. Materials

Poly(vinyl alcohol) (PVA, Warrington, PA, USA; the degree of saponification (DS) was 99.7 _mol_%, M_w_~78,000) and poly(acrylic acid) (PAA, M_w_~100,000); and poly(N,N-dimethylaminoethyl methacrylate) (pDMAEMA) with an intrinsic viscosity of 5 dL/g, which corresponds to MM > 1,000,000, and a degree of quaternization of 68 _mol_% (synthesized at the IMC RAS) were used in the experiments.

The multi-wall carbon nanotubes (MWCNTs) used in this work were functionalized with carboxylic groups according to the technique described in [43].

A commercial UPM-20 polyamide ultrafilter was supplied by Vladipor Inc. (Vladipor, Russia).

#### 2.1.1. Film Preparation

**Figure 2:**
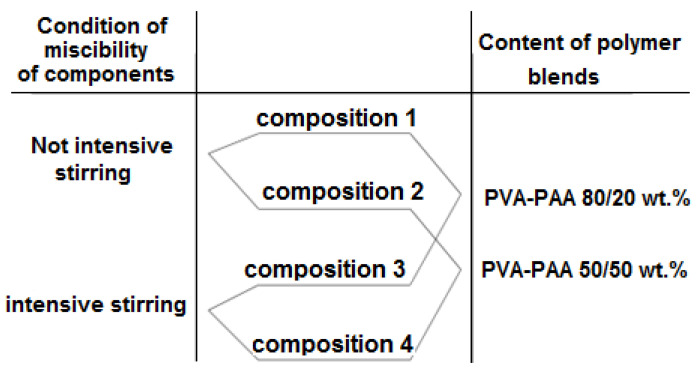
Preparation conditions for PVA-PAA compositions.

For the manufacture of the membranes, 6 wt.% solutions of PAA, PVA, and PAA-PVA mixtures were prepared. The composite materials were prepared by mixing aqueous solutions of the above polymers in two modes (compositions 1, 2 and 3, 4 were produced at less and more intensive stirring rates (800 and 1400 rpm, respectively) (Figure 2). The mixtures contained components in various ratios (the mixtures used in preparing compositions 1, 3 and 2, 4 contained 20 and 50 wt.% of PAA, respectively). Non-porous films were obtained from working (formation) solutions via the removal of the solvent under heating (110 °C) and subsequent cooling to room temperature.

Thus, the films were produced at temperatures that exceeded the glass transition temperatures of PVA and PAA but lay below the temperature range of the thermal esterification reactions (>125 °C) characteristic of these polymers [17]. The absence of intermolecular interactions with the formation of ester bonds both in thick (~20 µm) and thin (~2 µm) layers of composites was controlled using transmission IR spectroscopy and ATR-IR spectroscopy.

#### 2.1.2. Preparation of Film Membranes

Film membranes with a thickness range of 18–30 μm were formed by pouring dilute aqueous solutions (working/formation solutions) of polymers or their mixtures (PVA, PVA-PAA, PVA–pDMAEMA) onto a smooth flat surface according to the technique described below.

Based on the given film thickness (*l*), the mass of the polymer sample was determined by the following formula:

M_memb_ = ρ · S · *l* (1)

where ρ is the polymer density in g/cm^3^, S is the surface area in cm^2^, and *l* is the film thickness in cm.

The calculated amount of the filtered polymer solution was poured with a pipette onto a smooth surface. The samples were dried until a constant weight was reached at room temperature and then at 50 °C under reduced pressure.

#### 2.1.3. Preparation of Bilayer Composite Membranes

The pervaporation composite membranes were prepared by pouring dilute polymer solutions onto the porous (average pore diameter less than 200 Å) surface of a UPM-20 commercial polyamide ultrafilter (manufactured by Vladipor Inc., Vladimir, Russia). In this way, a non-porous diffusion layer was formed. Three types of diffusion membranes were prepared from the PAA-PVA mixture containing polymers in the 80/20 ratio. The membrane of the first type was prepared via the single casting of the PAA-PVA solution, the membrane of second type was obtained via double casting, and the membrane of the third type was prepared via single casting followed by a post-treatment (annealing at T = 110 °C for 30 min).

The membranes were produced from the 6 wt.% PAA-PVA solution containing the components in the 80/20 ratio. This concentration was chosen due to the fact that it is difficult to achieve the uniform deposition of a layer of polymer mixture on a substrate at concentrations exceeding 6 wt.%; on the other hand, at lower concentrations, coating microdefects are possible.

#### 2.1.4. Preparation of MMM-Type Membranes

Samples of nanocomposite membranes (membranes of the MMM type) were obtained via the introduction of MWCNT into solutions of polymer mixtures in the amount of 1 wt% of the total mass of all polymers. The dispersions were used for casting film membranes obtained in conditions in which heterogeneous membranes were received based on PAA-PVA mixtures.

### 2.2. Methods

#### 2.2.1. X-ray Diffraction (XRD) Analysis

The structure of samples was investigated with an automated DRON-2.0 X-ray diffractometer (SPA “Burevestnik”, Moscow, Russia), whereby the CuK_α_ radiation (wavelength λ = 1.5406 Å) filtered through the Ni foil was used. The measurements were carried out in the transmittance mode.

An X-ray diffraction (XRD) analysis of composites containing carbon nanotubes was performed at room temperature with the aid of a SEIFERT XRD 3003 TT diffractometer (GE, Sensing & Inspection Technologies GmbH, Wunstorf, Germany) equipped with a primary monochromator (U = 40 kV, I = 40 mA). The CuK_α_ radiation (wavelength λ = 1.5406 Å) was used. The X-ray diffraction patterns were obtained with a step of 0.05°; the scanning time was 10 sec at each point of the scattering angle range (2° < 2θ < 40°). The values of characteristic interplanar distances were calculated using Bragg’s equation. The interpolation of the obtained diffraction patterns was performed using moving average optimization followed by least-square polynomial interpolation.

#### 2.2.2. Atomic Force Microscopy (AFM)

Atomic force microscopy studies were carried out using a Nanotop NT-206 atomic force microscope (ODO “Microtestmachines”, Minsk, Belarus) in the tapping mode under atmospheric conditions (NSC11/AlBS silicon cantilevers, force constant = 1.5–5 N/m, tip curvature radius = 10 nm). The experimental data were processed using the Surface Explorer program.

#### 2.2.3. Differential Scanning Calorimetry (DSC) and Thermal Gravimetric Analysis

Differential scanning calorimetry experiments were performed with a 204 F1 differential scanning calorimeter (Erich NETZSCH GmbH & Co. Holding KG, Selb, Germany) in an argon atmosphere (argon flow rate: 25 mL·min^−1^; heating rate: 10 deg·min^−1^).

#### 2.2.4. IR Spectroscopy

Fourier transform infrared (FTIR) spectra were recorded with the aid of a Bruker Vertex 70 IR-Fourier spectrometer (Bruker Optik GmbH, Ettlingen, Germany) in the attenuated total reflection (ATR) mode (resolution: 4 cm^−1^; number of scans: 60). The spectrometer was equipped with a “Pike” attenuated total internal reflection microattachment with a ZnSe working element. During the registration of the FTIR spectra, a correction was made that took into account the dependence of the wavelength on the radiation penetration depth. 

In order to identify possible interchain crosslinks that appeared due to the formation of ester bonds, PVA-PAA films of various compositions subjected to post-treatment at temperatures ranging from 70 °C to 110 °C were also studied using IR spectroscopy in the transmission mode.

### 2.3. Pervaporation Experiments

#### 2.3.1. Vacuum Pervaporation

The pervaporation (PV) experiments were carried out with the help of the autonomous laboratory setup described in our previous publication (see the illustration in [44]). The setup consisted of the following components:

(i) A thermostatically controlled steel membrane module (a non-continuous water-thermostated PV cell equipped with the mixing device that provided the uniform density of the feed mixture). The working surface area of the membrane was 6.07 × 10^−4^ m^2^;
(ii) A vacuum pump, with which the permeate was removed by vacuum degassing followed by condensation in a liquid-nitrogen-cooled trap;
(iii) A receiver for collecting the permeate cooled with liquid nitrogen ensured the complete condensation of the vapors that passed through the membrane;
(iv) A pressure gauge that allowed for controlling the pressure under the membrane (3–5 mm Hg).

The selective transport properties of the studied samples in the separation of methanol/toluene and toluene/heptane mixtures were examined.

The main membrane characteristics (the flux through the membrane (J), the separation factor (F), and the penetration–separation index (PSI)) were determined from the weight gain in the ampoule and the obtained percentages of the components of the separated mixtures during the experiment.

The permeate was weighed, and the total flux J (kg m^−2^ h^−1^) through the membrane was calculated according to the following formula:

J = P/*l* = m·S^−2^ × t^−1^, (2)

where P is the flux normalized to membrane thickness *l* and m is the mass of the substance that has passed through the membrane area S in time t.

The separation factor f for binary mixtures was calculated using the following formula:

f_A_ = (Y_A_/Y_B_)/(X_A_/X_B_), (3)

where X_A_ and Y_A_ are the concentrations (wt.%) of the more penetrating component A in the initial mixture and permeate, respectively; X_B_ and Y_B_ are the concentrations (wt.%) of the less penetrating component B in the initial mixture and permeate, respectively.

#### 2.3.2. Analysis of Compositions of Liquid Mixtures

##### Refractometry

The values of the refractive index n_D_ for the studied mixtures of liquids were determined with an IRF-22 refractometer at a constant temperature of 25 °C. Before the measurements, the calibration dependences of refractive index values were obtained using toluene/n-heptane mixtures of the given compositions. The calibration plot and the measured values of refractive index were used to determine the toluene concentration in the permeate.

##### Chromatography

In addition to the refractometric method, the compositions of the permeates were determined via gas chromatography. An analytical column with Chromosorb P sorbent was prepared. The sorbent was elutriated, the moisture was removed, and the sorbent was dried at 150 °C for 4 h. The sorbent was coated with a stationary phase (TCEP, 1,2,3-tris-(2-cyanoethoxy)propane)) taken in an amount of 2 wt.% with respect to the sorbent weight. The chromatograms of the permeates were obtained under the following conditions: the evaporator temperature was 130 °C, the column temperature was 80 °C, and the flow rate of the carrier gas was 30 mL/min. 

The parameters of the chromatograms were used to calculate the contents of the components in the permeate. The calculations were carried out using the internal standard method. A known amount of the internal standard (propanol-1) was added to a weighed portion of the analyte, giving a well-resolved peak in the chromatogram.

The concentration of the determined component *C_i_* (wt.%) in the analyzed mixture was calculated using the following formula:

(4) $$C_{i}=\frac{S_{i}\;\cdot \;K_{i}\;\cdot \;M_{st}}{S_{st}\;\cdot \;M_{n}}\;\cdot \;100$$

where *S_st_* is the peak area of propanol-1; *K_i_* is the calibration factor; *M_st_* is the mass of added propanol-1; *M_n_* is the mass of the sample of the analyzed mixture, to which a certain amount of propanol-1 is added.

Using the internal standard method, the corresponding calculations for toluene/n-heptane mixtures of various compositions were performed. The calibration coefficients were used to calculate the percentages of these components in the permeate using the internal normalization method.

In this method, the sum of the areas of all peaks is taken as 100%, and the concentration of any component of the sample is calculated as the relative peak area:

(5) $$C_{i}=\frac{S_{i}}{\sum_{i=1}^{n}S_{i}}\;\cdot \;100$$

The calculation is carried out taking into account the calibration coefficients:

(6) $$C_{i}=\frac{S_{i}\;\cdot \;K_{i}}{\sum_{i=1}^{n}\left(S_{i}\;\cdot \;K_{i}\right)}\;\cdot \;100$$

where *C_i_* is the concentration of component *i* as a %; *S_i_* is the area of the corresponding peak; *K_i_* is the calibration factor; $\sum_{i=1}^{n}(S_{i}\;\cdot \;K_{i})$ is the sum of the products of peak areas calculated using relative correction factors for all peaks in the chromatogram.

The internal normalization method is convenient because it does not require a reproducible sample injection (in terms of the size and identity of the analysis conditions). The calculations are carried out using the relative calibration coefficients, which are not very sensitive to small changes in the experimental conditions.

## 3. Results and Discussion

### 3.1. The Structures of Films Based on PVA, PAA, and PVA-PAA Blends

A series of composite materials (compositions 1–4) was prepared from mixed solutions of polyvinyl alcohol and polyacrylic acid. Two techniques involving the combination of aqueous solutions of these polymers were used; composition 1.2 was prepared in mixing mode 1, and composition 3.4 was prepared in mixing mode 2. The component ratios were also varied; the working (formation) solutions for the preparation of compositions 1.3 and 2.4 contained 20 and 50 wt.% of PAA, respectively. The film formation process was carried out at temperatures exceeding the glass transition temperatures of each of the components, but not reaching the temperature range of the thermal esterification reactions (>125 °C) characteristic of these polymers.

The self-supporting film prepared from the 80/20 mixture of PVA and PAA was studied using IR spectroscopy. The FT-IR spectra of the individual polymers and the spectrum of the 80/20 PVA-PAA film heated to 110 °C were also obtained.

**Figure 3:**
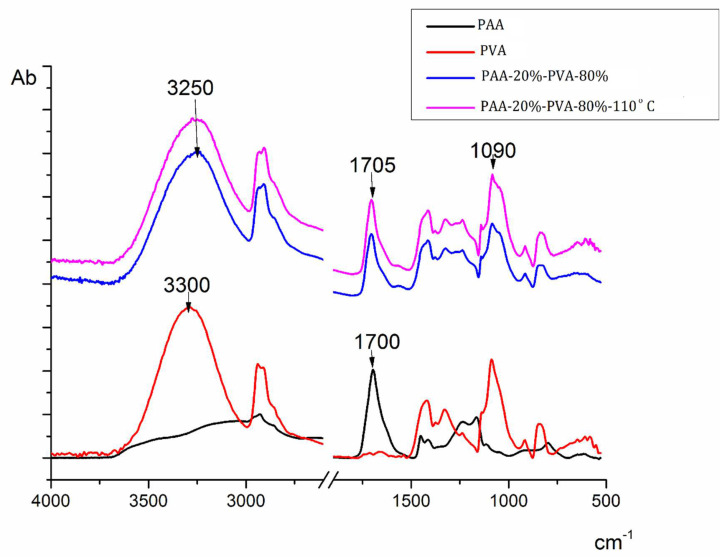
FTIR spectra of PVA-PAA samples.

Figure 3 displays the IR spectra of the PVA-PAA films taken before and after the thermal treatment at 110 °C for 30 min.

The above analysis of the spectra reveals a change in the position of the absorption band related to OH stretching vibrations (pure poly(vinyl alcohol) 3300 cm^−1^, pure poly(acrylic acid) 3000 cm^−1^, PVA-PAA mixture 3250 cm^−1^). The vibration band of the C=O group of PAA shifts from 1700 to 1705 cm^−1^. There are also changes in the shapes of the peaks located in the 1050–1100 cm^−1^ region, namely the band attributed to OH bending vibrations and the peak corresponding to stretching vibrations of the C-O bond in alcohol. However, even upon heating films up to 110 °C, no bands appear in the region of 1750–1735 cm^–1^, which is characteristic of the C=O stretching vibrations of the ester group. Thus, the IR spectroscopy data indicate the formation of hydrogen bonds between the components and the absence of covalent interactions in thermally treated samples.

The IR spectra contain no bands characteristic of ester bonds. Figure 3 shows that interactions with the formation of ester bonds appear neither in thick (20 µm) nor in thin (from 2 to 6 µm) layers of composites.

**Figure 4:**
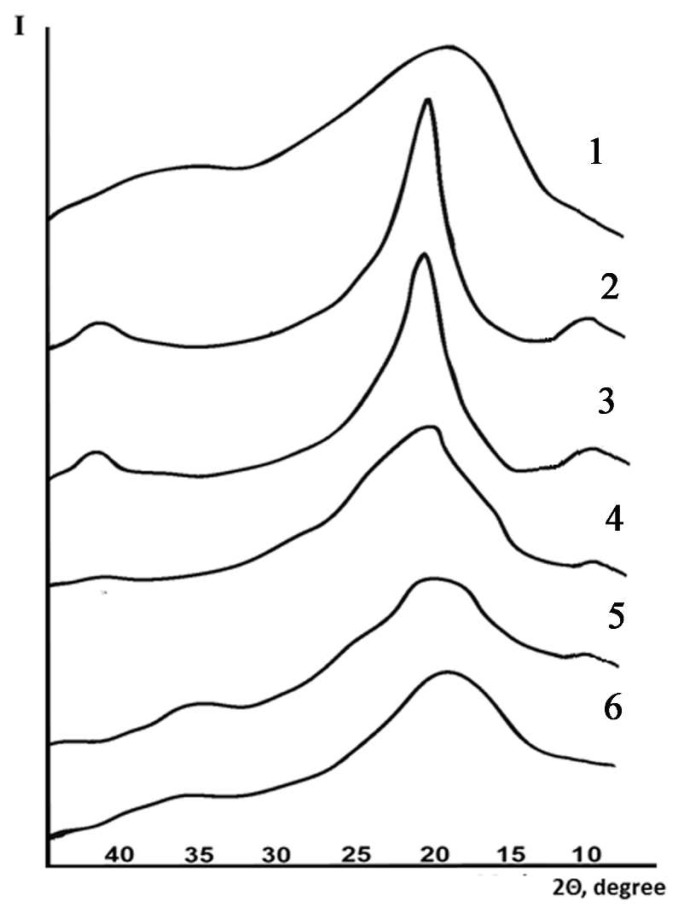
X-ray diffraction patterns of films prepared from PAA (curve 1), PVA (curve 2), and compositions 1–4 (curves 3–6, respectively).

Figure 4 presents the diffraction patterns of the PVA and PAA films, as well as the compositions on the basis of these two polymers.

The diffraction pattern of a PVA film contains a number of reflections at 2θ angles of 10°, 19°30’, and 41°, which characterize its crystalline structure. In the case of composition 1 containing 20 wt.% of PAA, the diffraction pattern practically does not differ from that of pristine PVA, while the composition can be considered a mixture of PVA and PAA. The introduction of 50 wt.% of PAA into polyvinyl alcohol (composition 2) leads to sample amorphization; the diffraction pattern contains two amorphous halos at the 2θ angles of 8° and 19°30’. The PAA diffraction pattern also exhibits an amorphous halo near 2θ = 19°. The amorphization can be observed in samples 3 and 4 (Figure 4), in which the PVA-PAA ratios (wt/wt) are equal to 80/20 (sample 3) and 50/50 (sample 4). This result indicates that the compositions are almost completely homogeneous. This conclusion is also confirmed by the DSC data; namely, only one glass transition temperature can be observed for each composition (3 and 4, see Table 1).

**Figure 5:**
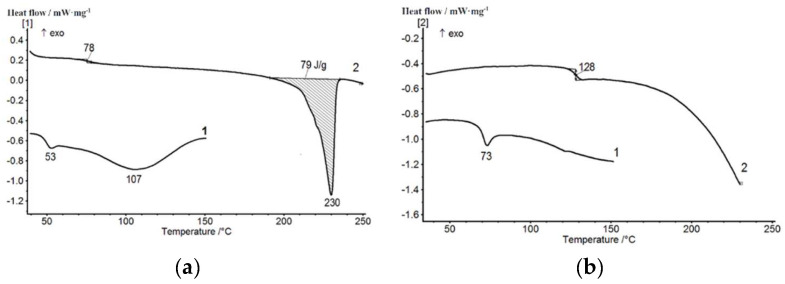
DSC curves of the first and second scans of PVA (**a**) and PAA (**b**) samples.

**Figure 6:**
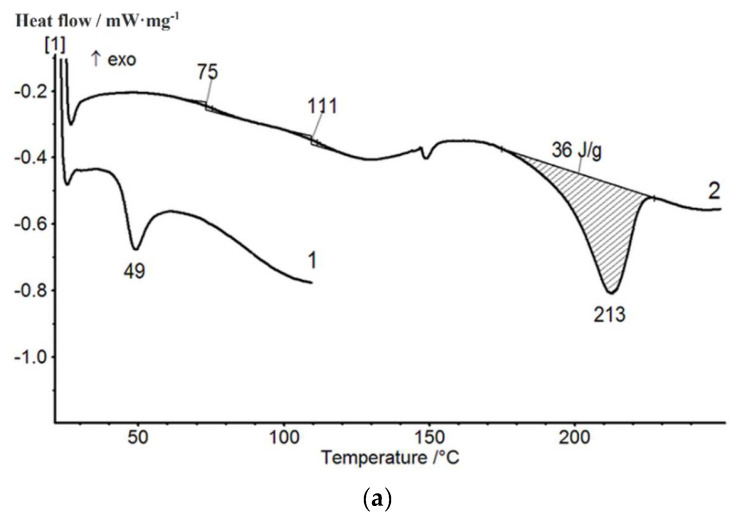

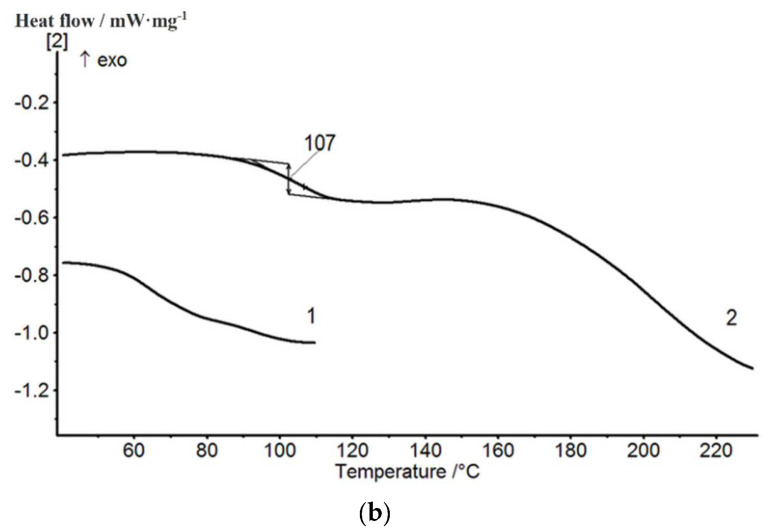
First and second DSC scans of PVA-PAA compositions: (**a**)—composition 1 (mode 1: mixing temperature 40 °C, low stirring speed, interaction time 40 min); (**b**)—composition 3 (mode 2: mixing temperature 40 °C, high stirring speed, interaction time 3 h).

The results of the thermophysical DSC studies of pristine polymers and their mixtures containing 20 wt.% of PVA (compositions 1 and 3 obtained in different mixing modes) are shown in Figure 5 and Figure 6. The first scans of the PVA and PAA samples were carried out at up to 150 °C in order to remove adsorbed water (Figure 5a). The second scan of a PVA sample showed the glass transition temperature of 78 °C and a melting temperature of 230 °C. The glass transition temperature of the PAA sample recorded during the second scan was 128 °C. The results obtained were in good agreement with the data reported in [36].

Figure 6 presents thermograms of the first and second scans of compositions containing 20 wt.% of PAA obtained in different mixing modes. The first scan was carried out at up to 110 °C; then, the samples were cooled down to room temperature and the second scan was performed. The results of the second scan were taken into consideration in the same way as in [45,46]. As can be seen, during the second scan in composition 1 (Figure 6a, curve 2), two glass transition temperatures were recorded (75 and 111 °C), which corresponded to the two phases of the mixture; then, melting of the crystalline PVA phase was observed at 213 °C. The difference between the enthalpy of melting of the crystalline phase in composition 1 (Figure 6a) and the enthalpy of melting of pure PVA (Figure 5a) can be explained by the different degrees of crystallinity of the PVA in these samples.

The first and second scans of composition 3 presented in Figure 6b indicate the homogenization of the mixture. In the second scan, only one glass transition temperature (107 °C) was recorded, with its value lying between the values of the glass transition temperatures of the initial components (see Table 1). One may conclude that at the same PAA content, the structure of the prepared PVA-PAA compositions (homogeneous or heterogeneous) depends on the selected mixing mode.

**Figure 7:**
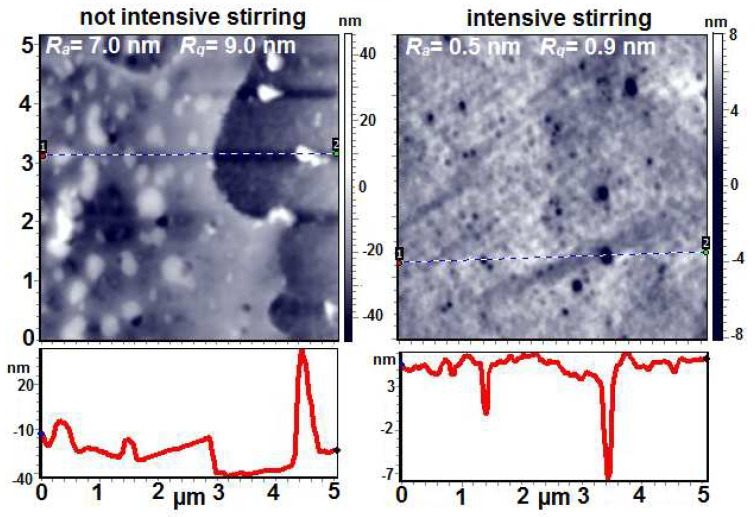
AFM height images and line profiles of PVA-PAA 80/20 (composition 1) and PVA-PAA 80/20 (composition 3).

The PVA-PAA compositions with the same mass ratio of components (80/20) obtained with low-intensity stirring and intensive stirring were studied using AFM in tapping mode (Figure 7).

In the case of the sample prepared with low-intensity stirring, the pattern of phase separation can be clearly observed on the “to the air” surface. The inclusions of the second phase are visible; their sizes vary from 50 to 400 nm, and their height above the average surface level reaches 40 nm. Nanopores with a diameter range of 50–150 nm and a depth range of 4–20 nm can also be observed on the surface of the film. The values of the arithmetic mean surface roughness R_a_ and root mean square surface roughness R_q_ for the scanning matrix measuring 5 × 5 microns in area are equal to 7.0 nm and 9.0 nm, respectively.

In the case of the composite obtained under intensive stirring, the surface of the film is noticeably smoothed and the roughness values decrease by an order of magnitude (R_a_ = 0.5 nm, R_q_ = 0.9 nm). A large number of pores (wells) can be observed, which are both very small (with a diameter range of 50–80 nm and a depth of no more than 4 nm) and relatively large (almost 10 nm deep, with diameters reaching 300 nm). It is interesting to note that due to the smoothing of the film surface, its fine structure can be seen; namely, the tangles of the macromolecules, which are well structured and arranged in mutually perpendicular directions, are exposed on the surface.

### 3.2. Pervaporation Properties of PVA, PAA, and Membranes Prepared from Their Blends

The processes of pervaporation involving non-porous films based on the studied polymers and their compositions were investigated. 

It should be noted that the separation of toluene-containing liquid mixtures via organophilic pervaporation is an urgent problem, which will be difficult to solve. The best known example is the isolation of alcohols from azeotropic mixtures with toluene, such as the mixture of methanol and toluene containing 29 wt.% of toluene [36]. As shown in Table 2, this mixture can be effectively separated using membranes with diffusion layers on the basis of PVA-PAA compositions. The selective membranes described in the scientific literature contain from 5 to 50 wt.% of PVA. Our studies demonstrated that polymer films containing significantly higher amounts of PVA (up to 80 wt.%) are equally efficient in the separation of this mixture. As for toluene/heptane mixtures, the efficiency of membranes with PVA-PAA diffusion layers containing 50–80 wt.% of PVA (composition 1) in separating toluene from its mixtures with heptane is on a level with the best known membranes used in this process.

**Figure 8:**
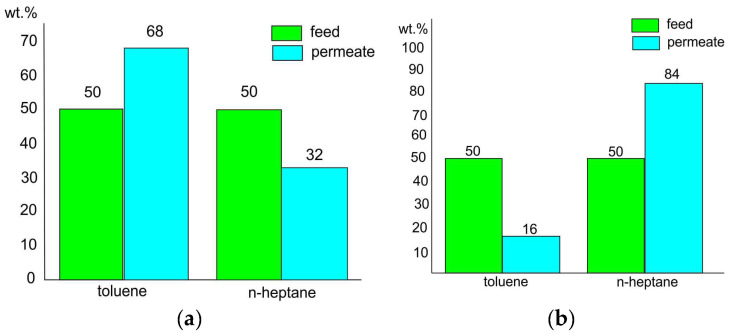
Comparison of transport properties of non-porous pervaporation membranes based on PVA-PAA mixture: composition 1 (**a**) and composition 3 (**b**).

The data presented in Table 2 and Figure 8 demonstrate the difference between the transport properties of homopolymers and composite materials prepared according to two techniques (two mixing modes, compositions 1 and 2). In addition, when the toluene/*n*-heptane mixture containing 50 wt.% of toluene is separated at 40 °C, the toluene concentration in the permeate increases to 68 wt.% for the membrane based on composition 1 and decreases to 16 wt.% for the membrane based on composition 3 (Figure 8). Thus, it may be concluded that depending on the conditions used during membrane preparation (mixing of components), it is possible to form composite materials that differ fundamentally in their mechanism of pervaporation transfer of toluene and n-heptane. Taking into account the peculiarities of the transport of mixtures of aromatic and aliphatic hydrocarbons that are discussed in the Introduction, we can assume that for homopolymers and composition 1, separation occurs predominantly according to the diffusion mechanism (toluene release). On the contrary, in the case of composition 3, the sorption mechanism predominates (isolation of n-heptane). These results indirectly indicate the difference in the structural organizations of composite materials 1 and 3.

Note that the X-ray phase analysis, differential scanning calorimetry, and IR spectroscopy studies of compositions 1 and 3 revealed that the differences between these samples relate to the presence of the continuous PVA phase in the former.

The difference in the pervaporation properties of compositions 1 and 3 can be explained by the formation of continuous domains of ordered PVA, which occurs during the preparation of a microheterogeneous film from the polymer mixture. It is known [48] that the areas with increased ordering, in which swelling is limited, in turn limit the swelling of the surrounding second phase and act as a diffusion barrier for low molecular weight liquids. Therefore, the transport of a substance occurs through the region consisting of the second more permeable polymeric component. Apparently, the formation of a continuous PVA phase prevents the swelling of the second component (PAA) to some extent and allows one to create a “transport zone” that determines the permeability and separation characteristics of the membrane as a whole.

### 3.3. Structure and Pervaporation Properties of PVA, pDMAEMA, and the Membranes Prepared from Their Blends

The hypothesis about the appearance of a “transport zone” (provided by the microheterogeneous structure of the membrane diffusion layer) requires verification. To this end, another permeable polymer should be used, while the domains consisting of the continuous PVA phase should be preserved. In other words, it is necessary to replace PAA with another polymer that meets the requirements taken into account in choosing PAA. In this case, the mechanism, and consequently the direction of separation, will remain the same. Based on the analysis of the literature data, poly-N,N-dimethylaminoethyl methacrylate (pDMAEMA) was selected as a second component for PVA-containing pervaporation membranes.

#### 3.3.1. Structural Features

Membranes based on poly-N,N-dimethylaminoethyl methacrylate (pDMAEMA) (gel immobilized on a porous inorganic substrate) have previously been successfully used to separate benzene/cyclohexane mixtures [49,50]. It was decided to test the above assumption about the formation of a certain “transport zone” due to the microheterogeneous structure of the diffusion layer of the membrane. Membranes based on PVA–pDMAEMA compositions were prepared using the same procedure as for the PVA-PAA compositions discussed above, and their thermophysical and transport properties were studied. 

**Figure 9:**
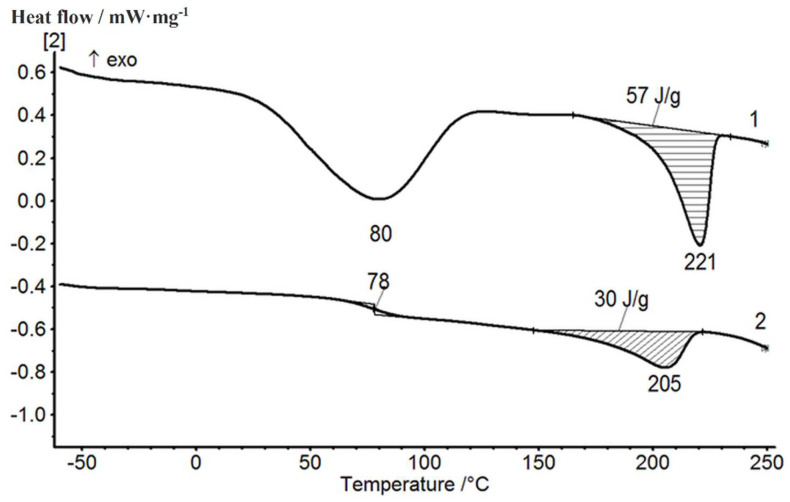
First and second DSC scans of the 90/10 (wt./wt.) PVA–pDMAEMA composition.

The DSC studies of the PVA-pDMAEMA composition containing the components in the 90/10 (wt./wt.) ratio (Figure 9) indicate that this film includes ordered PVA regions, and similarly to composition 1 (PVA-PAA), a microheterogeneous structure is formed.

**Figure 10:**
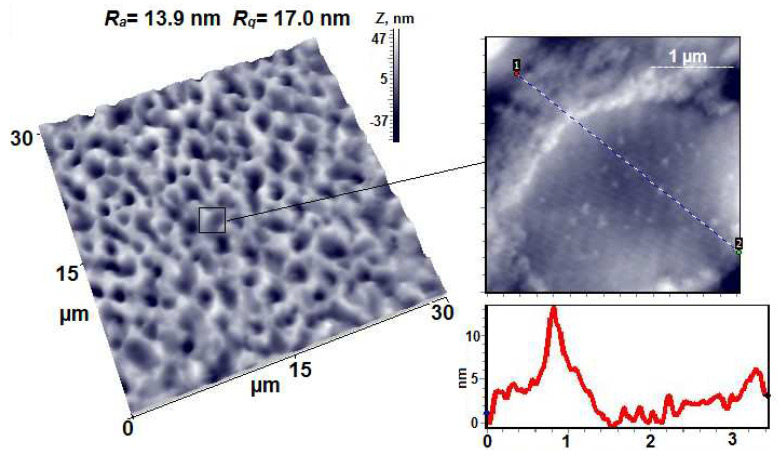
AFM 3D and height images and a line profile of PVA–pDMAEMA 90/10.

Figure 10 shows AFM images of the highly porous 90/10 PVA–pDMAEMA membrane. Numerous large pores and caverns with a size of 0.5–3 microns can be observed on the surface of the membrane. The pore depth reaches maximum values of ~100 nm, while the cavern depth does not exceed 20 nm. The surface roughness values R_a_ and R_q_ for the 30 × 30 µm scanning matrix are equal to 13.9 nm and 17.0 nm, respectively. An image of a cavern with a depth of ~ 5 nm taken at high magnification is also presented; the inclusions of the second phase are visible on its surface.

#### 3.3.2. Transport Properties of the PVA–pDMAEMA Composite Membranes

**Figure 11:**
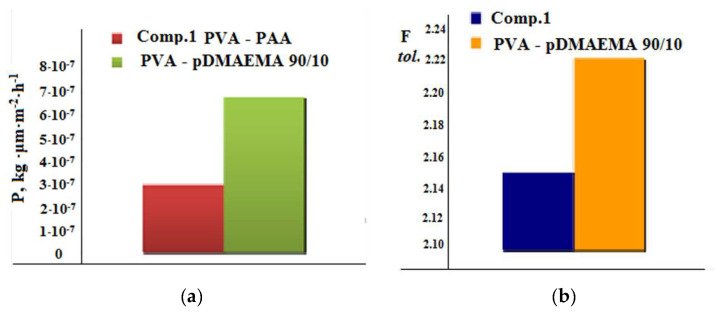
Comparison of the transport characteristics of the membranes based on the 80/20 PVA-PAA blend (composition 1) and the 90/10 PVA-pDMAEMA blend in the separation of the toluene/n-heptane mixture: (**a**) P is the permeability in kg·μm·m^−2^·h^−1^; (**b**) F is the separation factor.

Figure 11a,b illustrates the comparison of the transport characteristics of the membranes based on the 80/20 (wt./wt.) PVA-PAA blend (composition 1) and the 90/10 (wt./wt.) PVA-pDMAEMA blend. Figure 11a also shows a diagram displaying the change in the value of the flux through the membrane. Figure 11b presents a diagram of the change in the separation factor.

It can be seen from the diagrams that substituting PAA in composition 1 for pDMAEMA allowed us to improve the transport characteristics of the resulting pervaporation membrane, i.e., to increase the values of the flow through the membrane and the separation factor. Meanwhile, the direction of the selective mass transfer is the same for the two studied membranes (PVA-PAA, composition 1, and PVA-pDMAEMA). Apparently, the transport mechanisms are similar in both cases. This result substantiates the earlier assumption that the microheterogeneous structure of the pervaporation membrane determines its efficiency and the direction of separation.

### 3.4. Formation of More Permeable Composite Membranes with Diffusion Layers Based on PVA-PAA

The data discussed above show that the formation of microheterogeneous diffusion layers from polymer mixtures based on PVA leads to a significant increase in the selectivity of the pervaporation membranes. However, film membranes based on PVA-containing mixtures have low permeability for the target components. To improve the transport properties of the membranes with diffusion layers of this type, two methods were used: (1) the deposition of diffusion layers from polymer mixtures onto porous substrates; (2) the formation of mixed matrix membranes (MMM). Based on the literature data and the results of our previous investigations, we can estimate the advantages of these approaches. The first method helps reduce the thickness of the diffusion layer and contributes to the efficiency of the separation of mixtures of liquids considerably differing in polarity (toluene/methanol). The second method improves the separation of mixtures of liquids that have similar properties but differ in their ability to interact with a less densely packed component of the heterogeneous polymer mixture (toluene–n-heptane).

#### 3.4.1. Composite Membranes with the PVA-PAA Diffusion Layers on a Porous Support

These composite membranes were obtained via the deposition of diffusion layers of various thicknesses on the upper surface of an UPM-20 asymmetric ultrafiltration polysulfonamide membrane (the average diameter of the pores on the working surface was less than 20 nm).

The data given in Table 3 show that the two-layer membranes with diffusion layers consisting of composition 1 on the UPM porous support are much more permeable than the similar films without supports. At the same time, the membranes are highly selective in the separation of azeotropic mixtures (29 wt.% of toluene).

#### 3.4.2. Composite Membranes of the MMM Type

In recent years, a large number of publications have been devoted to the modification of organo-inorganic composite membranes by introducing various nanofillers, including carbon nanotubes [51].

**Figure 12:**
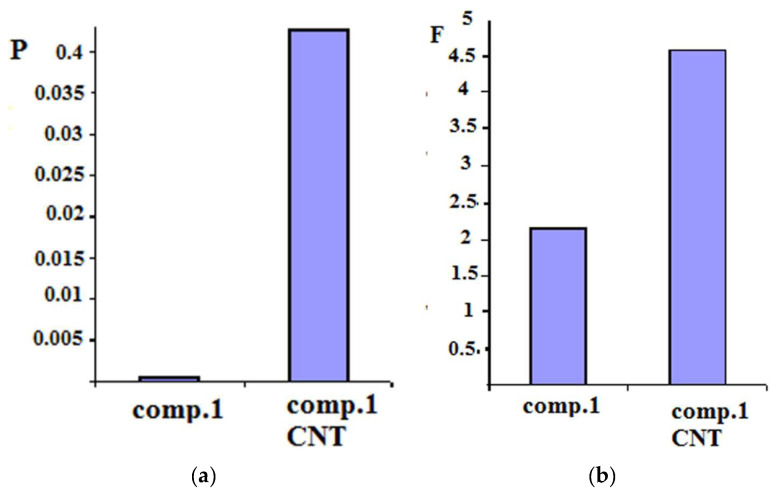
Comparison of the flux normalized to the membrane thickness P [kg μm m^−2^ h^−1^] (**a**) and the separation factor f (**b**) for membranes based on the PVA-PAA mixture and PVA-PAA with MWCNT (1 wt.%) while separating the 50/50 (wt./wt.) toluene/n-heptane mixture.

In this work, two types of multi-walled carbon nanotubes (MWCNTs) (pristine and with treated surfaces) were introduced into composition 1 at extremely low concentrations. As a result, the transport properties of this membrane changed significantly. Thus, in the case of “untreated” CNTs, the separation factor for the PVA-PAA membrane (Figure 12b) increased more than two-fold, while the permeability increased by an order of magnitude (Figure 12a).

**Figure 13:**
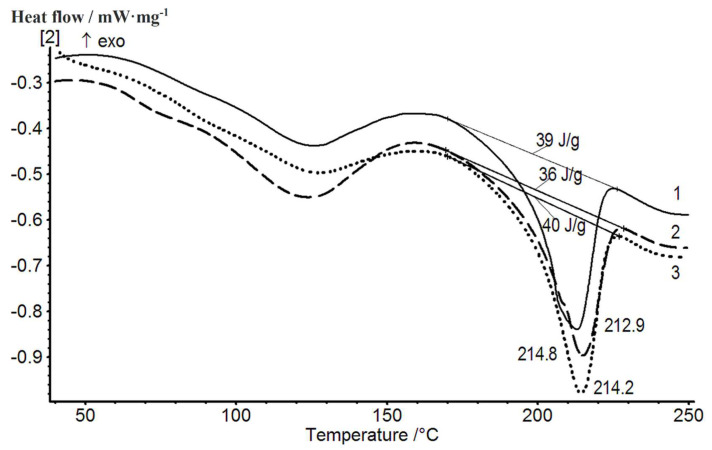
Thermograms of samples of the 80/20 PVA-PAA compositions obtained in mode 1 (composition 1): 1—the original composition; 2—composition containing 1 wt.% of carbon NTs with surface carboxyl groups; 3—composition containing 1 wt.% of untreated carbon NTs.

The structure and thermal properties of composition 1 containing pristine and treated carbon nanotubes were studied using DSC and wide-angle X-ray scattering. The results of the second scan of these samples are shown in Figure 13. The DSC data demonstrate a slight increase in the melting temperature of the film containing nanotubes (as compared to that of the pristine composition), while the enthalpies of melting of the crystalline phase practically do not change (Figure 13).

**Figure 14:**
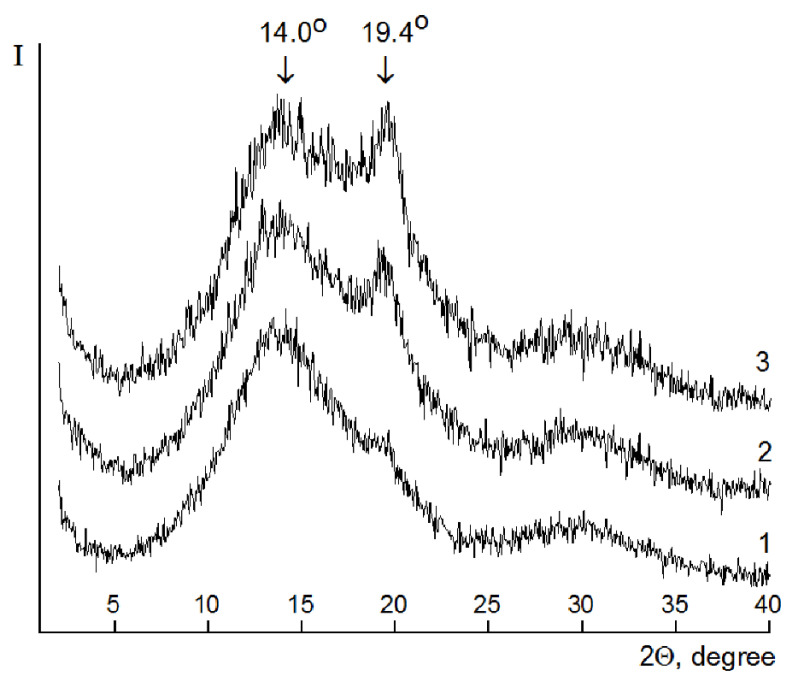
X-ray diffraction patterns of the 80/20 PVA-PAA compositions obtained according to mode 1 (composition 1): 1—original composition; 2—composition 1 containing 1 wt.% of untreated carbon NTs; 3—composition 1 containing 1 wt.% of carbon NTs with surface carboxyl groups.

The X-ray phase analysis of the PVA-PAA samples (composition 1) and PVA-PAA nanocomposites (composition 1 containing carbon nanotubes) revealed an exfoliated distribution of nanoparticles in the PVA-PAA mixture. No reflection at a scattering angle 2θ = 25.9° (d = 0.343 Å) characteristic of the graphite structure was observed. The obtained diffraction patterns contained two peaks at 2θ values of 14.0° (d = 6.3 Å) and 19.4° (d = 4.6 Å) (Figure 14, curves 1–3). The introduction of nanotubes into the polymer composition led to the rearrangement of the reflections and increased their intensity. It can be assumed that the degree of crystallinity of the nanotube-containing sample was higher than that of the pristine PVA-PAA composition (Figure 14 curves 1–3).

The data obtained indicate that carbon nanotubes introduced into a heterogeneous PVA-PAA blend in small amounts (up to 1 wt.%) are well dispersed in the polymer matrix, which exerts an influence on the structure of this binary polymer composition. The number of ordered polymer domains with low permeability increases, and the transport of liquids preferably occurs in the areas where carbon nanotubes are located. Because nanotubes are capable of strong sorption interactions with the aromatic component of the separated mixture, NTs contribute to the preferential transport of toluene through the pervaporation membrane.

## 4. Conclusions

The composite membranes intended for the separation of liquid organic mixtures were prepared on the basis of polyvinyl-alcohol-containing polymer blends. It was revealed that the processes occurring during the removal of a solvent from the concentrated working or formation solution (containing a mixture of two polymers) exert a decisive influence on the structure of the prepared film. Depending on the degree of homogenization of these concentrated solutions, microheterogeneous or homogeneous polymeric films can be formed. The system of hydrogen bonds in the films, or more precisely the presence of unbound polar groups, and the presence of the continuous crystalline PVA phase determine the functional properties of the produced membranes. Therefore, non-porous polymer films of various structures (with different types and degrees of ordering) can be obtained from the same polymers taken in the same quantitative ratios in solutions of the same concentrations; it is only necessary to slightly change the preparation conditions. The observed structural effects can become more pronounced if the second component of a polymer mixture is purposefully selected (in our case, pDMAEMA instead of PAA) or a nano-sized filler that can be well dispersed in the polymer matrix is introduced. By changing the factors that determine the nature of mutual distribution of the two polymeric components, it is possible to control the mechanism of liquid transport through a composite polymer film. Namely, this allows one to change the predominantly transported component in the process of selective transfer through a diffusion membrane.

## Figures and Tables

**Table 1:** Glass transition temperatures of PVA, PAA, and their compositions.

Polymer	Glass Transition Temperature (DSC Data), °C
PVA	78.0
PAA	128.0
Composition 1 (20 wt.% PAA)	75.4; 111.4
Composition 2 (50 wt.% PAA)	115.2
Composition 3 (20 wt.% PAA)	106.7
Composition 4 (50 wt.% PAA)	112.3

**Table 2:** Separation characteristics of the membranes obtained in the present work and their analogues described in the literature.

N	Membrane	Components of the Feed Mixture	T, °C	The Amount of Toluene, wt.%		Refs
				Feed Mixture	Permeate	
1	Poly(oxyethylene methacrylate)	Toluene—n-heptane	80	21	56	[47]
2	PVA-PAA (10–40 wt.% PVA)	methanol-toluene	25	29	~5	[36]
3	PVA-PAA (80 wt.% PVA) Composition 1	Toluene—n-heptane	40	50	68	“this work”
4	PVA-PAA (80 wt.% PVA) Composition 1	methanol-toluene	40	29	2.5	“this work”

**Table 3:** Pervaporation properties of (PVA-PAA 80/20)/UPM-20 membranes.

Membrane, N	Composition of the Feed Mixture, wt.%		T, °C	Permeate, C(methanol), wt.%	J, kg m^−2^ h^−1^	f	Number of Coating Procedures	Post—Treatment, T, °C
	Methanol	Toluene						
1	100	0	22	100	0.28 ± 0.01	-	1	-
1	70	30	22	78.70	2.25 ± 0.11	1.58	1	-
1	50	50	22	56.35	0.92 ± 0.05	1.29	1	-
1	0	100	22	0	0.020 ± 0.001	-	1	-
2	71	29	22	99.07	0.080 ± 0.004	21.29	2	-
2	71	29	40	97.43	0.120 ± 0.006	10.79	2	-
2	67	33	40	97.82	0.59 ± 0.03	12.95	2	-
3	71	29	22	97.72	0.180 ± 0.009	12.95	1	110

## Data Availability

Not applicable.

## Abbreviations

**PVA**	poly(vinyl alcohol)
**PAA**	poly(acrylic acid)
**pDMAEMA**	poly(N,Ndimethylaminoethyl methacrylate)
**XRD**	X-ray diffraction
**AFM**	atomic force microscopy
**DSC**	differential scanning calorimetry
**ATR IR**	attenuated total reflection infrared spectra
**PV**	pervaporation
**DS**	the degree of saponification
